# Target-AID-Mediated Multiplex Base Editing in Porcine Fibroblasts

**DOI:** 10.3390/ani11123570

**Published:** 2021-12-16

**Authors:** Soo-Young Yum, Goo Jang, Okjae Koo

**Affiliations:** 1 Department of Veterinary Clinical Science, College of Veterinary Medicine, Seoul National University, Seoul 08826, Korea; sy.yum@lartbio.com (S.-Y.Y.); snujang@snu.ac.kr (G.J.); 2 ToolGen, Inc., Seoul 08501, Korea

**Keywords:** Target-AID, porcine, pig, base editing, CRISPR/Cas

## Abstract

**Simple Summary:**

CRISPR/Cas9 driven multiplex genome editing may induce genotoxicity and chromosomal rearrangements due to DNA double-strand breaks at multiple loci simultaneously. To overcome this problem in porcine cells we utilized Target-AID, a base editing system, to edit multiple loci in the porcine genome. We showed that the Target-AID system works well in porcine fibroblasts with up to 63.15% efficiency. This is the first report demonstrating that the Target-AID system works well in porcine cells and can be used to generate genome-edited pigs.

**Abstract:**

Multiplex genome editing may induce genotoxicity and chromosomal rearrangements due to double-strand DNA breaks at multiple loci simultaneously induced by programmable nucleases, including CRISPR/Cas9. However, recently developed base-editing systems can directly substitute target sequences without double-strand breaks. Thus, the base-editing system is expected to be a safer method for multiplex genome-editing platforms for livestock. Target-AID is a base editing system composed of PmCDA1, a cytidine deaminase from sea lampreys, fused to Cas9 nickase. It can be used to substitute cytosine for thymine in 3–5 base editing windows 18 bases upstream of the protospacer-adjacent motif site. In the current study, we demonstrated Target-AID-mediated base editing in porcine cells for the first time. We targeted multiple loci in the porcine genome using the Target-AID system and successfully induced target-specific base substitutions with up to 63.15% efficiency. This system can be used for the further production of various genome-engineered pigs.

## 1. Introduction

The development of programmable nucleases, including the clustered regularly interspaced short palindromic repeats (CRISPR)/CRISPR-associated protein (Cas) system, has significantly increased the efficiency of genetic engineering of livestock and has ushered in a new era in animal biotechnology. This technology can be used to enhance livestock traits, such as improved meat production [1] or disease resistance [2], generate precise disease models of human diseases [3], produce donor organs for xenotransplantation [4], and improve animal welfare [5].

Conventional genome-editing systems, such as CRISPR/Cas9, rely on DNA repair mechanisms after target-specific double-strand breaks (DSBs) in DNA. Although these systems are very efficient, they are limited in their use as a multiplex genome engineering tools because simultaneous DSBs at multiple loci induce genotoxicity and chromosomal rearrangements [6]. This disadvantage of conventional genome-editing systems for multiplex genome engineering can be overcome using the base-editing system. The base editing system involves the fusion of the CRISPR/Cas nickase with a deaminase, such as rAPOBEC [7] or PmCDA1 [8], which can substitute DNA base pairs at target sites without DSBs. Thus, base editing is expected to be a safer method for multiplex genome-editing platforms for livestock. However, base editing also has limitations. The base-editing system can only substitute a target base within the “editing window”, which is specified by the Cas or deaminase enzymes used in the system. To overcome this limitation, several different base editors with different editing windows have been developed to expand the available target sites for base editing [9].

Target-AID is a base-editing system composed of PmCDA1 and a cytidine deaminase from sea lampreys fused to Cas9 nickase [8]. The Target-AID system can substitute cytosine for thymine in 3–5 base editing windows at 18 bases upstream of the protospacer-adjacent motif (PAM) of Cas9 nickase. The Target-AID system was first developed and validated in CHO cells [8] and was then validated in plants [10,11,12,13], fungi [14], yeast [15,16], bacteria [17], and zebrafish [18,19] cells. However, to the best of our knowledge, there are no reports describing the use of the Target-AID system in livestock, particularly in pigs.

In the current study, we demonstrated Target-AID-mediated base editing in porcine cells for the first time. We targeted multiple loci in the *gag* and *pol* genes of porcine endogenous retrovirus (PERV) in the porcine genome using the Target-AID system and successfully induced target-specific base substitution.

## 2. Materials and Methods

### 2.1. Design of Guide RNA Sequences

The complete genome sequences for PERV-A, PERV-B, and PERV-C were obtained from GenBank (accession numbers KY484771, AY099324, and HM159246, respectively). All of the guide RNA (gRNA) sequences for the Target-AID systems were designed using BE-Designer tools on the CRISPR RGEN Tools website (http://www.rgenome.net/be-designer/, accessed on 12 December 2021). First, we designed a series of gRNAs targeting the *gag*, *pol*, and *env* genes for each of PERV-A, PERV-B, and PERV-C. We then selected gRNAs that induced a premature stop codon for each gene. Next, we selected single gRNAs that could target the same region on all the PERV-A, PERV-B, and PERV-C genes simultaneously. Finally, we selected the gRNA sequences, 5′-ttcaggttaagaagggacct-3′ for *gag* and 5′-acagtaccccttgagtagag-3′ for *pol,* to use for further experiments (Table 1). 

### 2.2. Vector Construction

**Figure 1:**
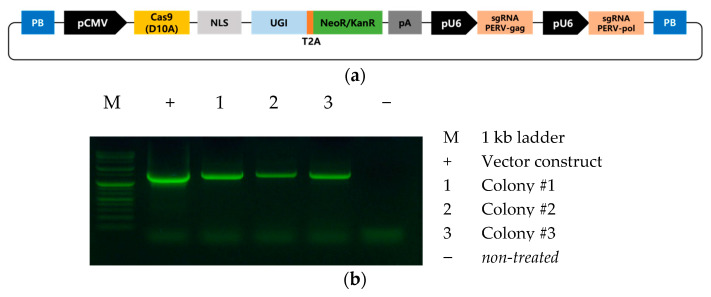
Target-AID vector system used in the study. (**a**) Scheme of the PB-CMV-Target-AID-PERV(*pol*-*gag*) vector used in this study. (**b**) PCR analysis of the target AID vector integration in porcine fibroblasts.

The vector for the Target-AID system was obtained from Addgene (#79620; Watertown, MA, USA) and cloned into the *piggyBac* vector used in our previous studies [20,21] using MluI and NotI restriction enzymes. In addition, we added two *U6* promoter-driven single guide RNA sequences targeting the *gag* and *pol* genes, using the In-Fusion HD Cloning Kit (Takara Bio, Kusatsu, Japan). The scheme for the vector used in this study, PB-CMV-Target-AID-PERV(*pol-gag*), is shown in Figure 1a.

### 2.3. In Vitro Culture of Porcine Fibroblasts and Transfection

Immortalized porcine fibroblasts [22] were cultured in DMEM (Gibco, Waltham, MA, USA) supplemented with 1% penicillin/streptomycin (Gibco), 10% fetal bovine serum (Gibco), 100 mM β-mercaptoethanol (Sigma, St Louis, MO, USA), and 1% nonessential amino acids (Gibco) at 37 °C with 5% CO_2_. We transfected 3 × 10^5^ cells using the Neon Nucleofection system (Invitrogen, Carlsbad, CA, USA). Briefly, 500 ng of the PB-CMV-Target-AID-PERV(*pol-gag*) vector and 500 ng of the transposase vector (pCy43, provided by the Sanger Institute) were transfected at 1400 V for 20 ms, with a pulse number of 2. After 2 d of transfection, the fibroblasts were treated with 2 µg/mL neomycin (Sigma) for 10 d for antibiotic selection. After selection, single cells were subcultured in each well of 96-well plates and then expanded. Cell colonies from each single cell were analyzed by PCR to confirm the integration of the vector. The primer sequences are listed in Table 2.

### 2.4. Targeted Deep Sequencing

The on-target regions of the gRNAs were PCR-amplified from genomic DNA extracted from transfected cells using Phusion polymerase (New England BioLabs, Ipswich, MA, USA). The resulting amplicons were subjected to paired-end deep sequencing using a Mi-Seq instrument (Illumina, San Diego, CA, USA). Deep sequencing data were analyzed using the online BE-Analyzer tool (http://www.rgenome.net/be-analyzer/, accessed on 12 December 2021). C-to-T base substitutions in the editing window, 16 to 19 bp upstream of the PAM sequence, were considered to result from the Target-AID system. The primers used in this study are listed in Table 2.

## 3. Results and Discussion

In the current study, we selected PERV integrated into the porcine genome as a target for base editing. PERV is a retrovirus that is found in most pig genomes in multiple copies. It is a potential risk in pig-to-human xenotransplantation, because most related retroviruses, such as human immunodeficiency virus, induce severe diseases, including immunodeficiencies [23] in host animals. However, because PERV is integrated into the porcine genome, it is impossible to remove this virus from pigs even when they are maintained in a microorganism-barrier facility. In 2015, Yang et al. [24] reported the inactivation of 62 copies of PERV *pol* genes in the porcine genome using the CRISPR/Cas9 system. However, 2 years later, the same group reported genotoxicity and chromosomal abnormalities induced by simultaneous DNA cleavage at multiple PERV sites [25]. To avoid the risk of genotoxicity induced by multiplex DSBs we used a Target-AID-based base-editing system to inactivate PERV in the porcine genome. We followed the CRISPR-STOP strategy used in a previous study [26], with slight modifications. Since the Target-AID system substitutes cytosine for thymine we carefully selected in-frame CAG, CAA, or CGA sequences in the open reading frame and designed gRNAs to change the sequences into the stop codons, TAG, TAA, or TGA, respectively. As a result of base editing, the target gene was knocked out due to the introduction of a premature stop codon.

Three PERV subtypes (PERV-A, PERV-B, and PERV-C) have been identified. Each PERV genome is composed of three core genes, *gag*, *pol*, and *env*. In this study, we attempted to design a single gRNA to simultaneously target all three PERV subtypes. As shown in Table 1, we designed two gRNAs simultaneously targeting the *gag* and *pol* genes of all three PERV subtypes. A gRNA for *env* was not used because we could not find a single gRNA targeting this gene in all PERV subtypes.

**Figure 2:**
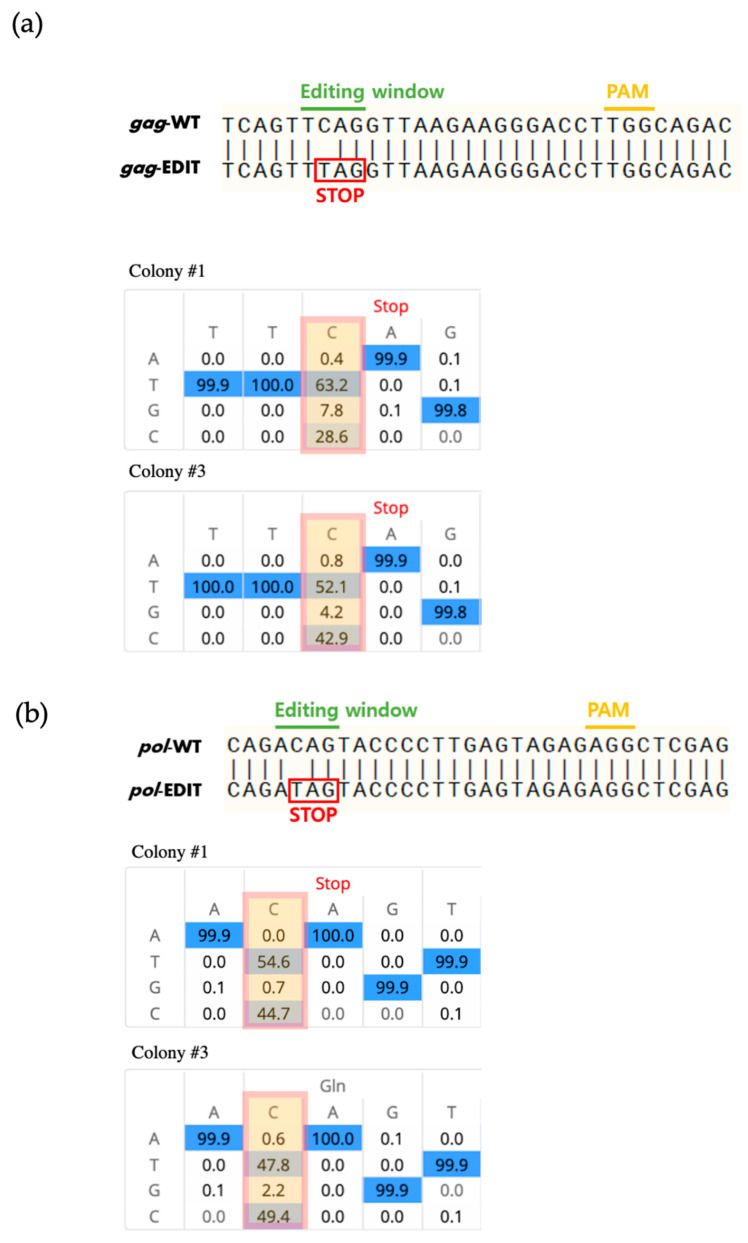
Base edited results at the target locus of Target-AID. (**a**) Sequence alignment of *gag* from wild-type and edited cell lines. (**b**) Sequence alignment of *pol* from wild-type and edited cell lines.

We constructed a *piggyBac*-based vector containing the Target-AID system with two sgRNAs targeting the *gag* and *pol* genes (Figure 1a). We transfected the vector into porcine fibroblasts and confirmed the integration of the vector by PCR analysis after 10 days of antibiotic selection. As expected, the transfected vector was successfully integrated into the genome of all tested cells (Figure 1b). To confirm the base-editing events mediated by the Target-AID system, targeted deep sequencing analysis was performed at the gRNA target site. We found successful C-to-T substitutions at the *gag* (Figure 2a) and *pol* genes (Figure 2b) in all tested cell colonies, whereas nontreated wild-type cells showed no substitutions (Table 3). Colonies #1 and #3 showed high substitution rates; however, colony #2 showed a relatively lower substitution rate (Figure 1b). A previous study in which the *pol* gene of PERV was targeted using a BE3 base editor [27] also failed to achieve 100% efficient removal of active PERV. This may be related to the integration site or the copy number of the Target-AID vector in the transgenic cells. Further studies are required to improve the efficiency of base editing in porcine cells. On the other hand, it might be possible that our PCR result for colony #2 is false positive due to plasmid DNA contamination. For this reason, we are focusing on sequencing data from colonies #1 and #3. Interestingly, we found that substitution at *gag* (63.15% and 52.12% for colonies #1 and #3, respectively) showed slightly higher efficiencies than substitution at *pol* (54.60% and 47.83% for colonies #1 and #3, respectively). Because both gRNAs were expressed from the same integrated vector, this result indicated that gRNA design also influences the efficiency of Target-AID-mediated base substitutions.

There are some previous reports of base editing in porcine cells using another base editing system, BE [27,28,29,30]. As the “editing windows” of the Target-AID and BE systems are different, these two base-editing systems may be used complementarily for complex porcine genome engineering. Furthermore, in combination with zygote electroporation [31] or somatic cell nuclear transfer [32], it is expected that base-edited pigs can be generated using the Target-AID system. Particularly, for generating PERV inactivated pigs, ribonucleoprotein (RNP) based Target-AID is recommended to avoid safety issue in clinical xenotransplantation. Genome-wide off-target studies for the Target-AID system are also recommended for the same reason. Further studies are required for adopting Target-AID based system in clinical conditions.

## 4. Conclusions

In conclusion, the current study showed, for the first time, that the Target-AID system can be used for base editing of multiple loci in porcine fibroblasts. We confirmed that the Target-AID system successfully induced a C-to-T substitution at the target site of the porcine genome with up to 63.15% efficiency. This system can be used for the further production of multiplex genome-engineered pigs.

## Figures and Tables

**Table 1:** Design of target gRNA sequences used in the study. Among the potential targets, the orange-highlighted gRNAs were selected for use in this study. PERV = porcine endogenous retrovirus. Cytosines in Editing Window marked in red letters indicate target sequences of Target-AID.

Target Gene	Direction	Stop Codon	CRISPR Target	Editing Window	Position	PERV-A	PERV-B	PERV-C
PERV *gag*	+	CAA	CCAACGCCTCACGGGGTTGGTGG	CAAC	705	o	o	o
	+	CAG	TTCAGGTTAAGAAGGGACCTTGG	TCAG	83	o	o	o
	+		GCAGACACTCTTCACAGCCGAGG	CAGA	780	x	x	x
	+		CCAGAAAGCCTCAGTGGCCCTGG	CAGA	1122	o	o	o
	+		TCAGAGACTGGAAGGGTTACAGG	CAGA	1185	o	o	o
	+	CGA	GCGAGAGAGAATTCTGTTAGAGG	CGAG	804	o	o	x
PERV *pol*	+	CAA	TCAAGATATACAGTCCTGGTTGG	CAAG	126	o	o	x
	+		CCCAAACCCTAGGACCATGGAGG	CCAA	1214	o	o	x
	+	CAG	ACAGTACCCCTTGAGTAGAGAGG	CAGT	255	o	o	o
	+		GACAGTACACCCTAGAAGACTGG	ACAG	2105	o	o	o
	+		CCAGTTCTCTGAGACTCCGGAGG	CAGT	2148	o	o	o
	+	CGA	AGCGATGGCTGACGGAGGCACGG	GCGA	899	o	o	o
	+		TCCGAGATTTGGAATACCTAAGG	CCGA	2582	x	o	o
	-	CCA	ACCAGTTCCGTTCAGGCGGGAGG	CCAG	483	o	o	x
	-	TCA	CTTCAGTTGAATAACCTGTGGGG	TTCA	206	o	o	x
	-	CTA	TTCTAAGCAGTCCTGTTTGGTGG	TCTA	761	o	o	o
	-		TTCTAGGGTGTACTGTCGTCTGG	TCTA	2099	o	o	o
PERV *env*	+	CAG	AACAGGAAAATATTCAAAAGTGG	ACAG	581	x	o	x
	+		ACCAGGGGTGGTTTGAAGGATGG	CCAG	1751	o	o	x
	+	CGA	CCGAGTGTACTACCATCCTGAGG	CGAG	1308	x	o	x
	-	CTA	GTCTATAAGGCGTTTACTACTGG	TCTA	122	x	o	x
	-	CCA	GACCATGACACAGAAATCTTTGG	ACCA	1274	x	o	x
	-		ACCATCCTTCAAACCACCCCTGG	CCAT	1752	o	o	x
	-		ACCCACTCGTTCTCTAACAAAGG	CCCA	1883	x	x	x
	-	TCA	CGTCAGAGCAGAAAGCAGGGTGG	GTCA	1796	o	o	x
	-	CTA	TCCTATGCATGTCCCCTTCCCGG	CCTA	1100	x	o	x

**Table 2:** Primer sequences used in the study.

Purpose	Strand	Sequence
Vector integration confirmation	F	5′-CCTCGTGCTTTACGGTATCG-3′
	R	5′-ATGCTCAAGGGGCTTCATGA-3′
PERV-*gag*	1st-F	5′-CTGGTGGTCTCCTACTGTCG-3′
	1st-R	5′-CTCCAAGAGCCAGGATTCGG-3′
	2nd-F	5′-GTCTTGTGCGTCCTTGTCTA-3′
	2nd-R	5′-CGTAAGGATATAGGGCTCCT-3′
PERV-*pol*	1st-F	5′-CCATCACTGTGTTGACCCTC-3′
	1st-R	5′-GGTGTAATCTCAGGCAGAAG-3′
	2nd-F	5′-TATACAGTCCTGGTTGGAGC-3′
	2nd-R	5′-ATTGACCTCTCTCAAGTCCT-3′

F, forward; R, reverse; PERV, porcine endogenous retrovirus.

**Table 3:** C-to-T substitution mediated by the Target-AID system in porcine fibroblasts.

Target Gene		C-to-T Substitution (%)
PERV-*gag*	*Nontreated*	0.22
	Colony #1	63.15
	Colony #2	1.65
	Colony #3	52.12
PERV-*pol*	*Nontreated*	0.11
	Colony #1	54.60
	Colony #2	1.61
	Colony #3	47.83

PERV, porcine endogenous retrovirus.

## Data Availability

The data that support the findings of this study are available from the corresponding author upon request.

## Abbreviations

AID	Activation-Induced Cytidine Deaminase
APOBEC	Apolipoprotein B mRNA Editing, Catalytic Polypeptide-like
BE	Base Editors
Cas9	CRISPR-associated protein 9
CHO	Chinese Hamster Ovary
CRISPR	Clustered Regularly Interspaced Short Palindromic Repeats
DMEM	Dulbecco’s Modified Eagle Medium
DNA	Deoxyribonucleic Acid
DSB	Double Strand Breaks
PAM	Protospacer Adjacent Motif
PCR	Polymerase Chain Reaction
PERV	Porcine Endogenous Retrovirus
PmCDA1	Petromyzon marinus Cytidine Deaminase 1
RNA	Ribonucleic Acid
